# Dehydration-induced earthquakes identified in a subducted oceanic slab beneath Vrancea, Romania

**DOI:** 10.1038/s41598-021-89601-w

**Published:** 2021-05-13

**Authors:** Thomas P. Ferrand, Elena F. Manea

**Affiliations:** 1 Institut des Sciences de la Terre d’Orléans, CNRS UMR 7327, Université d’Orléans, Orléans, France; 2 National Institute for Earth Physics, Calugareni, 12, Măgurele, Ilfov Romania; 3 GNS Science, PO Box 30-368, Lower Hutt, New Zealand

**Keywords:** Solid Earth sciences, Geology, Geophysics, Mineralogy, Seismology, Tectonics

## Abstract

Vrancea, Eastern Romania, presents a significant intermediate-depth seismicity, between 60 and 170 km depth, i.e. pressures from 2 to 6.5 GPa. A debate has been lasting for decades regarding the nature of the seismic volume, which could correspond to the remnant of a subducted slab of Tethyan lithosphere or a delamination of the Carpathians lithosphere. Here we compile the entire seismicity dataset (≈ 10,000 events with 2 ≤ Mw ≤ 7.9) beneath Vrancea for P > 0.55 GPa (> 20 km) since 1940 and estimate the pressure and temperature associated with each hypocenter. We infer the pressure and temperature, respectively, from a depth-pressure conversion and from the most recent tomography-based thermal model. Pressure–temperature diagrams show to what extent these hypocentral conditions match the thermodynamic stability limits for minerals typical of the uppermost mantle, oceanic crust and lower continental crust. The stability limits of lawsonite, chloritoid, serpentine and talc minerals show particularly good correlations. Overall, the destabilization of both mantle and crustal minerals could participate in explaining the observed seismicity, but mantle minerals appear more likely with more convincing correlations. Most hypocentral conditions match relatively well antigorite dehydration between 2 and 4.5 GPa; at higher pressures, the dehydration of the 10-Å phase provides the best fit. We demonstrate that the Vrancea intermediate-depth seismicity is evidence of the current dehydration of an oceanic slab beneath Romania. Our results are consistent with a recent rollback of a W-dipping oceanic slab, whose current location is explained by limited delamination of the continental Moesian lithosphere between the Tethyan suture zone and Vrancea.

## Introduction

**Figure 1:**
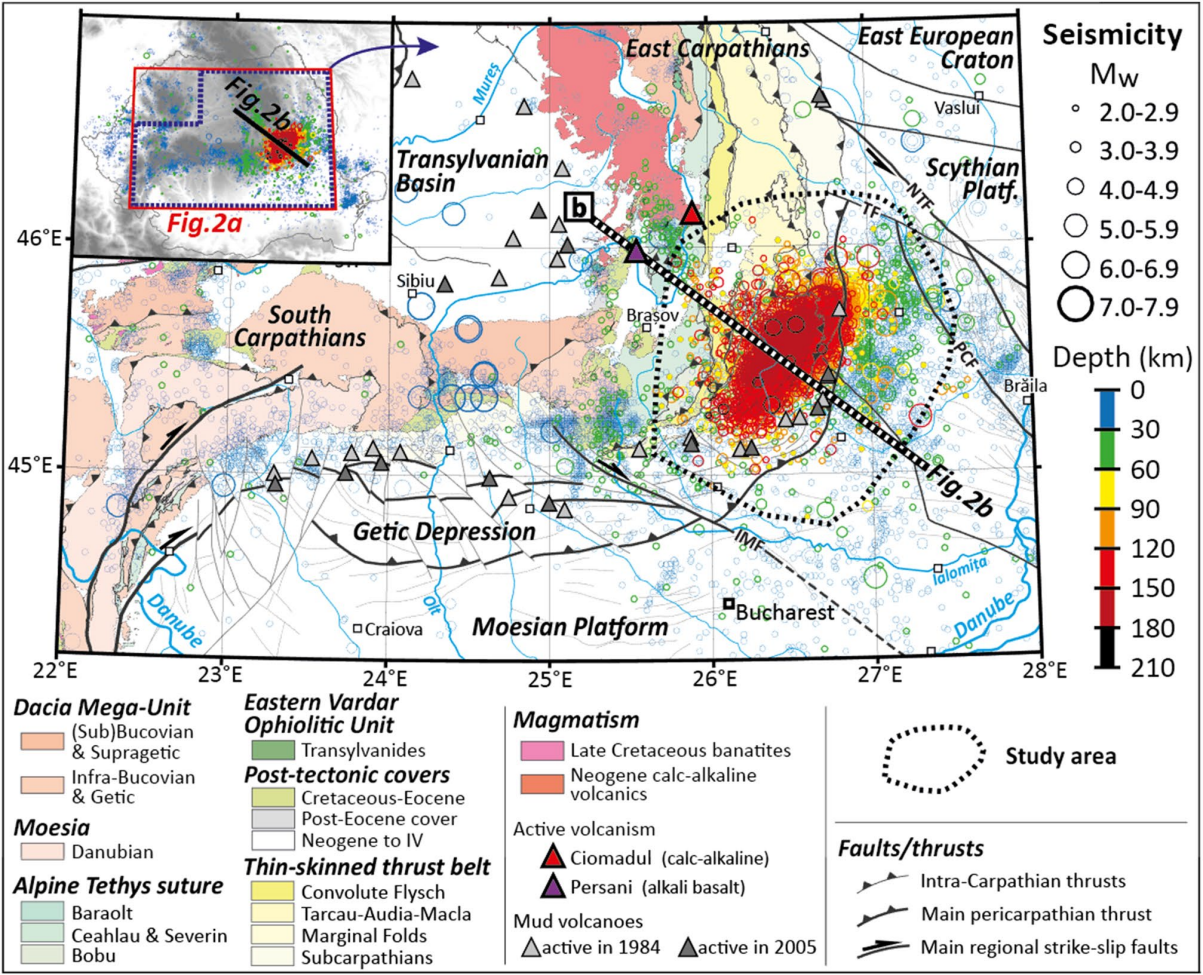
Intermediate-depth seismicity beneath Vrancea. (**a**) Synthetic geological map of central and eastern Romania, showing the location of epicentres for all seismic events (Mw ≥ 2) from 1940. Hypocentral depths are indicated with the colour scale. The study area is presented with the black contour. The locations of the 3D model (Fig. 2a) and interpreted profile (Fig. 2b) are shown on the top left inset. Map construction: tectonic features from Maţenco et al.^9^, epicentral locations from the *BIGSEES* and *ROMPLUS* catalogues; volcanoes location from Molnár et al.^37^, mud volcanoes from Paraschiv^58^ and Baciu and Etiope^33^.

**Figure 2:**
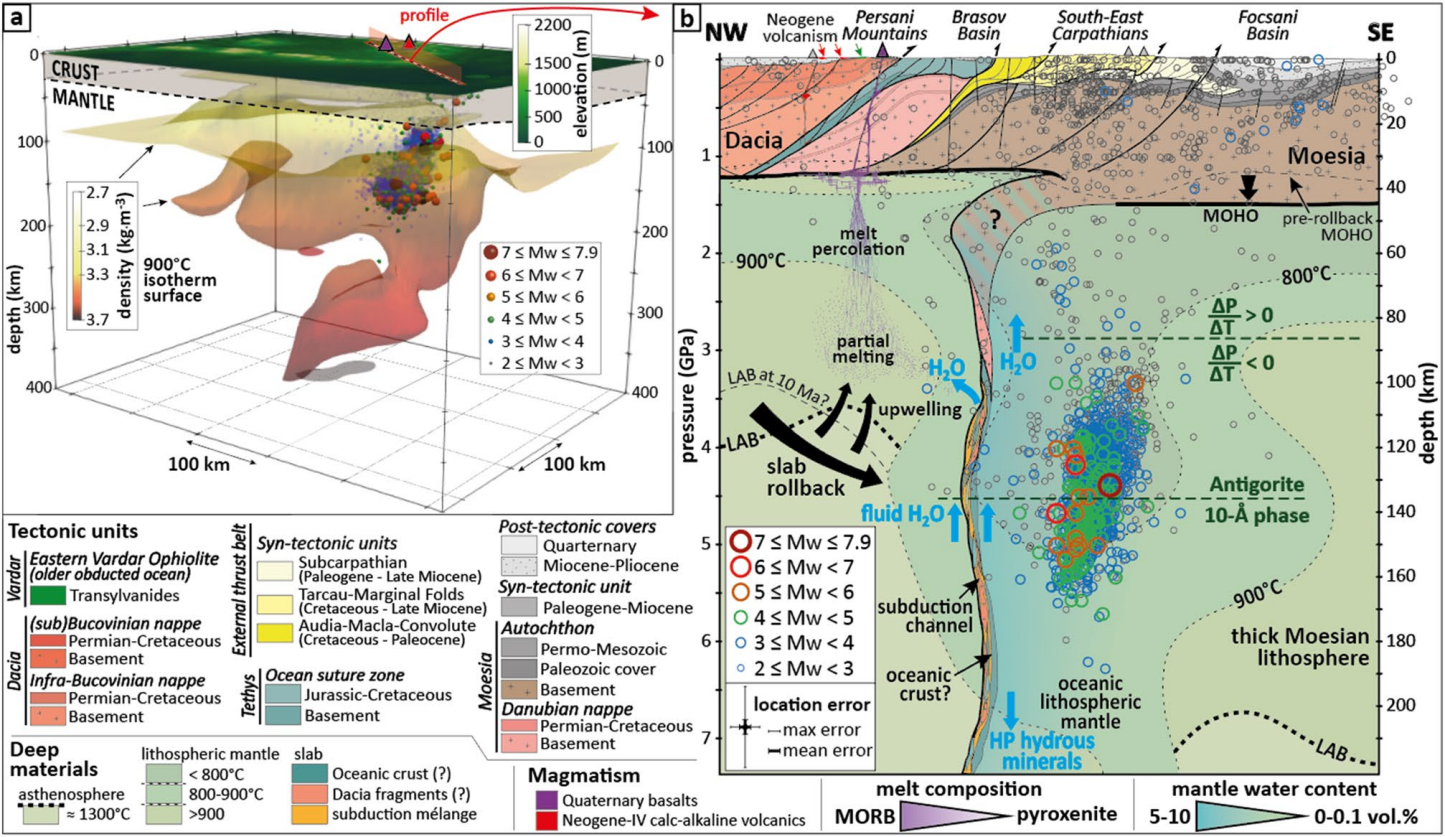
Geometry of the Vrancea slab and seismicity depth distribution. (**a**) 3D model showing the geometry of the slab, using the shape of the 900-°C isotherm extracted from the most recent thermal model^38^. Open circles show the hypocentre locations of the entire seismic dataset (Mw ≥ 2, year ≥ 1940). The location of the synthetic cross-section (**b**) as well as the locations of the PVF and Ciomadul are located on top of the 3D model (see text); (**b**) Interpreted cross-section of the Vrancea slab normal to the seismic body (scale 1:1). The profile from NW (46.2; 25.2) and SE (45; 27.3) is located on (**a**). All earthquakes shown on the cross-section are located at < 10 km from the profile. The position of the Lithosphere-Asthenosphere Boundary (LAB) is from Dérerová et al.^59^ The pre-rollback position of the Moho is from Maţenco et al.^9^, and the former LAB position (10 Ma) is proposed following the rollback scenario. The 800-°C and 900-°C isotherms are extracted from the thermal model. For profile location, see (**a**) and Fig. 1. The ΔP/ΔT refers to the Clapeyron slope of antigorite dehydration, which is positive for P < 2.8 GPa and negative for P > 2.8 GPa. Location uncertainties are represented on (**b**), with vertical and horizontal errors of 5.2 and 3.4 km, respectively.

The contact area between the Carpathians and the Moesian Platform (Fig. 1) is highly seismic^1–3^. In particular, and most surprisingly, the Vrancea region, located at the SE corner of the Carpathians, is characterized by a puzzling intermediate-depth high-seismicity body^4–6^, with the highest seismicity between 60 and 170 km depth (Fig. 2). The seismic body correlates with a near-vertical high-velocity mantle anisotropy^5^ and a negative temperature anomaly (Fig. 2a) deduced from seismic tomography^5^. A debate has lasted for decades regarding whether the Vrancea intermediate-depth seismicity is associated with the subduction of an oceanic slab or the delamination of the continental lithosphere^4,7–11^. A review of upper-mantle phases shows a correlation of most intermediate-depth seismicity with the instability of minerals in various subduction zones around the Earth^12^. In the present paper, after recalling the recent discoveries regarding transformation-induced seismicity (e.g.^13–15^), we apply the same correlation method to the Vrancea slab in order to decipher which phases are more likely the trigger of earthquakes in each P–T condition and deduce the nature of the Vrancea slab.

Intermediate-depth (30–300 km) and deep (300–750 km) earthquakes are a common feature of the solid Earth, the vast majority of which nucleate within subducting oceanic lithospheres^16,17^. Thermal models and precise hypocentre relocation show that temperature within sinking slabs is dominated by the advection of cold material, and that the depth to which seismicity extends is controlled by the lithosphere age and the subduction rate^16,17^. Experimental studies show a clear link between intermediate-depth seismicity and dehydration reactions in both the mantle^13^ and oceanic crust^15,18^. At intermediate-depth in subduction conditions, hydrous minerals are not metastable^19^ and their dehydration reactions are fast enough to trigger earthquakes^12,20^. Dehydration reactions are not necessarily seismogenic^13,14,18^, yet dehydration-driven stress transfers trigger earthquakes within slightly serpentinized subducting slabs^13^. These experimental findings have been further supported/complemented by seismological studies^12,21,22^, rupture nucleation simulations^23^ and field works showing that seismic faults develop in relatively dry bodies while hydrous rocks deform and dehydrate in a more homogeneous and aseismic manner^24,25^. A recent scaling law highlights the striking similarities between natural earthquakes and their experimental analogues^26^, providing additional validation for representativeness of the experimental results.

Minerals triggering earthquakes upon dehydration are the result of seawater seepage into the oceanic lithosphere before it enters subduction, especially along bending faults and reworked transform faults, which roots can reach the brittle-ductile transition^12,27^, e.g. 30–40 km depth for the old Pacific Plate offshore Japan or Mariana^22,28^. It is also possible that part of the hydration of the deep oceanic lithosphere comes from the mantle-scale water cycle (e.g.^29^) through underplating processes during plate formation (e.g.^30^).

When an oceanic lithosphere subducts below a continental lithosphere, dehydration fluids percolate through the subduction channel towards the upper plate, which induces calc-alkaline volcanism, frequently followed by alkaline volcanism once the subduction is over^31,32^. In a classic subduction setting, volcanism is a consequence of slab dehydration and water transfer to the upper plate. Large earthquakes in Romania are frequently accompanied by enhanced activity of mud volcanoes (located on Fig. 1), which are evidence of fluid pathways within the crust of both the Dacia and Moesia tectonic units^33^, suggesting that subduction volcanism, significant during the Neogene^32^, is limited by limited water transfer due to slab verticalization. Additionally, the SE Carpathian region is characterized by enhanced electrical conductivity that likely highlights enhanced water percolation within the suture zone of the Carpathian belt and surrounding areas^34,35^. In contrast, decompressional melting, which occurs in case of lithospheric delamination, results in basaltic volcanism^7,32^. The origin of the East Carpathians recent magmatism^3^ has been highly debated since there is no evidence of oceanic subduction in the area since the Miocene^7^. The Ciomadul volcano is located at the SE extremity of the Călimani-Gurghiu-Harghita volcanic chain (NW–SE line). Its last eruption is very recent (57–30 ka^36,37^) and geological and geophysical data together support that it is still active^3,7^. The volcanic chain has been active over the Neogene, with very last activity nowadays at the Ciomadul cone, while older volcanism (1.5 Ma) affected the Apuseni area^32^. The Persani Volcanic Field (PVF^7^ is offset ≈ 30 km westward from the calc-alkaline volcanic chain^32^. Delamination‐induced magmatism is expected to produce such basalts (^7^ and references therein). The eastward migration of subduction magmatism is fully consistent with the rollback of an oceanic slab between the Miocene and the Quaternary^7^, also supported by a detailed study of the eastward migration of both uplift and subsidence during this period^9^.

**Figure 3:**
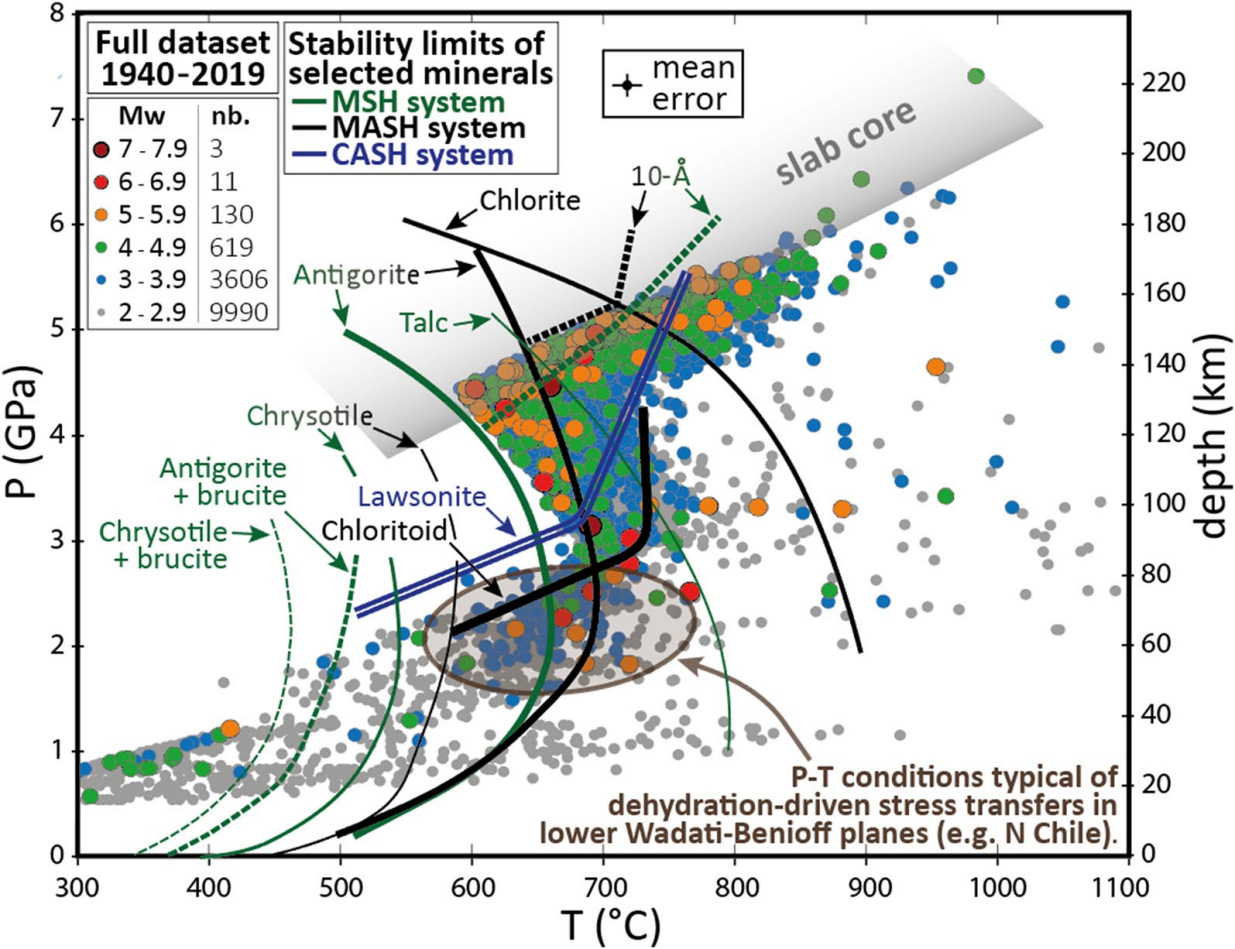
Pressure and temperature at hypocenters beneath Vrancea. P–T conditions at hypocenters from 20 to 240 km depth, highlighting the “S” shape of Vrancea intermediate-depth seismicity. At first order, three main trends are observed: (1) 30–80 km: P–T conditions consistent with relatively warm subducting oceanic mantle (lower Wadati-Benioff plane, N Chile^12^, (2) 80–170 km: P–T conditions consistent with serpentine dehydration (negative Clapeyron slope) in relatively warm subducting mantle (mostly antigorite) and (3) 170–240 km: P–T conditions consistent with the center (coldest part) of a relatively warm subducting slab, which could be consistent with the dehydration of either the 10-Å phase (Fig. 4a) and phase A (Fig. 4b), i.e. mantle, or lawsonite (Fig. 4c). For a detailed review of mineral destabilizations considering either mantle or crust, see Fig. 4. For chemical formula, see Table S1. Error bars indicate the mean uncertainties, which are 0.22 GPa in pressure (6.5 km in depth) and 25 °C in temperature. Abbreviations: MSH = MgO-SiO_2_-H_2_O; MASH = MgO-Al_2_O_3_-SiO_2_-H_2_O; CASH = CaO-Al_2_O_3_-SiO_2_-H_2_O.

**Figure 4:**
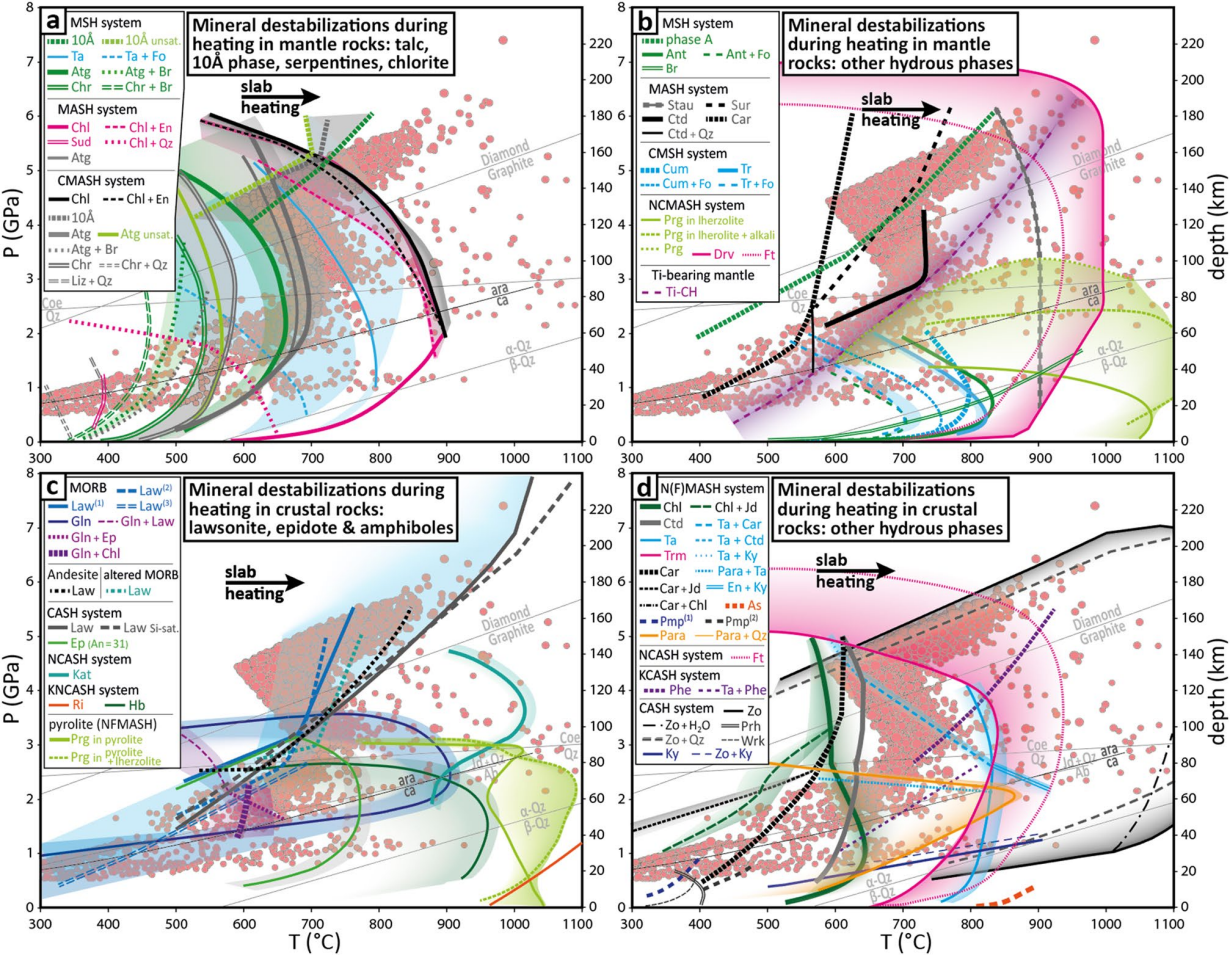
Vrancea deep seismicity and mineral destabilizations in the oceanic lithosphere. P–T conditions at hypocenters from 20 to 240 km depth compared to a review of mineral destabilizations in the mantle (**a**,**b**) and oceanic crust and subduction channel (**c-d**) during heating. For chemical formula and mineral reactions, see Table S1. Abbreviations: 10Å = 10-Å phase; A = phase A; Ab = albite; Ant = antophyllite; Atg = Antigorite; Ara = aragonite; As = aspidolite; Br = brucite; Ca = calcite; Car = carpholite; Ctd = chloritoid; Chl = chlorite; Chr = chrysotile; CH = clinohumite; Coe = coesite; Cum = cummingtonite; Drv = dravite; En = enstatite; Ep = epidote; Fo = forsterite; Ft: Mg-foitite; Gln = glaucophane; Hb = Hornblende; Jd = jadeite; Kat = Mg-Katophorite; Ky = kyanite; Law = lawsonite; Lz = lizardite; Para = paragonite; Phe = phengite; Phl = phlogopite; Pmp = pumpellyite; Prg = pargasite; Prh = prehnite; Qtz = quartz; Ri = K-richterite; Stau = staurolite; Sud = sudoite; Sur = Mg-sursassite; Ta = talc; Tr = tremolite; Trm = tourmaline; Wrk = Wairakite; Zo = zoisite. Chemical systems: MORB = Mid-Oceanic-Ridge Basalt; K = K_2_O; N = NaO; M = MgO; A = Al_2_O_3_; C = CaO; S = SiO_2_; H = H_2_O; F = OF_2_. Phases formula and references are presented in Table S1.

Vrancea intermediate-depth seismicity appears enigmatic and has puzzled seismologists for decades (e.g.^4,8–10^). Combining the most recent thermal model of Vrancea^38^ with the full dataset of hypocenters relocation (see theMethods), we infer the pressure and temperature for each event for depths > 20 km (Fig. 3), which we compare with the stability limits of hydrous minerals expected in continental and oceanic lithospheres. The inferred pressures vary from ≈ 0.5 to ≈ 7.5 GPa, and the temperatures from ≈ 100 to ≈ 1100 °C. A systematic review of the experimentally-deduced stability limits of hydrous phases (Fig. 4) shows contrasting results, with a surprisingly good correlation between most seismic events and the destabilization of antigorite and talc minerals (mantle materials), but also lawsonite (oceanic crust and/or subduction channel) and chloritoid (continental crust and/or metasomatized lithospheric mantle). Such correlations can always be discussed and controversial, due to multiple mineral candidates for a signal event, stability shifts due to compositional variability and uncertainties associated with P–T estimates^12^. Here, however, the S-shape P–T distribution (Fig. 3) demonstrates the existence of a dehydrating mantle slab beneath Vrancea.

For pressures from 1 to 4.5 GPa (35 to 135 km depth), most Vrancea events correlate with the destabilization of antigorite, which is the most stable hydrous phase in relatively cold mantle slabs in this pressure range^12^ and references therein). At higher pressures, Vrancea events correlate with the destabilization of the 10-Å phase, which is a high-pressure hydrated talc produced by high-pressure antigorite dehydration and is the most stable hydrous phase for pressures from 4.5 to 6.5 GPa (135 to 190 km depth^12^ and references therein). The three branches of the S-shape P–T distribution (Fig. 3) can be explained separately: (1) between 55 and 85 km depth, the hypocentres indicate typical conditions for mineral destabilizations in a slightly hydrated mantle, with the same P–T conditions as observed for the lower Wadati-Benioff plane of the Chilean subduction zone^12^, (2) between 85 and 135 km depth, the seismicity follows the negative Clapeyron slopes of antigorite and talc dehydration reactions; and (3) between 135 and 180 km depth, the seismicity correlates with the dehydration of the 10-Å phase.

When trying to investigate the link between the annual cumulative seismic moment and potential volume changes due to phase transformation, Ismail-Zadeh et al.^39^ found that volume changes are not sufficient to explain the intermediate-depth seismicity in Vrancea. This is consistent with experimental results evidencing that reactions volume changes are only a secondary parameter in the triggering mechanism of seismic events within peridotite samples at intermediate depths^13^. In other words, mineral destabilizations are not the process that releases energy, but the trigger of a process that releases energy, i.e. seismic ruptures.

It should be recalled that rupture propagation can be highly asymmetric in certain conditions (e.g.^40^, which means that the hypocentre depth (centre of energy radiation) can vary from the rupture nucleation depth (initial mechanical instability) and the transformation causing the rupture. In this study, we assume, consistently with recent numerical simulations on the largest recorded strike-slip earthquake (Mw 8.6, Indian Ocean, 2012^23^) that the hypocentre and nucleation locations are the same.

Several lines of evidence support the existence of an oceanic slab beneath Vrancea: (1) earthquakes hypocenters in stable continental settings are limited to depths where T < 600 °C^10^, (2) the regional tectonic reconstruction of the Carpathians include this oceanic subduction in the northern and eastern Carpathians^11^, (3) recent/active subduction volcanism is observed in the area^3,32^ and (4) as detailed in the present study, a strong correlation exists between the intermediate-depth seismicity and the dehydration of serpentine and talc minerals, which suggests that a slab of oceanic mantle is currently dehydrating beneath Romania. Numerous studies (e.g.^10,41^) suggested that subcontinental seismic zones such as the Vrancea slab were produced by the subduction of oceanic lithosphere. Nevertheless, even considering the verticalization of the slab during the Quaternary^7^, the location of the Vrancea seismic zone is offset ≈ 100 km to the East compared to the Tethyan suture zone^42^ (Figs. 1 and 2b). No trace of any additional subduction can be found in the Focşani Basin right above the seismic body^9^. Although the absence of evidence is not evidence of absence, as McKenzie et al.^10^ aptly recalled, it is always puzzling when the subduction leaves so little evidence of its former existence (^10^ and references therein). Hence, one could interpret the Vrancea slab as an example of intracontinental subduction^43^ involving decoupling and subduction of the lower continental lithosphere in a collisional context^42^.

Continental subduction is well documented thanks to the exhumation of UHP rocks in several orogens^44,45^. These rocks reached > 80 km depth (> 2.7 GPa^44^) and continental material has even been speculated to reach depths greater than 200 km^46^. As geologists have failed to find evidence of an oceanic suture zone in the Vrancea region, it has been speculated that the vertical slab, which reaches the mantle transition zone (> 410 km; Fig. 2), would consist of the delamination of the continental lithosphere^10^. Our results rather suggest that the continental material in the Vrancea slab reaches only 80–90 km depth and is due to limited continental delamination that has occurred since the Miocene due to slab pull. Sheared fragments of the Dacia continental lithosphere are also likely within the subduction channel (Fig. 2b), but the latter is by nature very thin and discontinuous.

According to the correlations we present between Vrancea intermediate-depth seismicity and mineral instability (Figs. 3 and 4), the nest of intermediate-depth earthquakes beneath Romania would correspond to a sub-vertical slab of oceanic lithosphere. The slab contains hydrous minerals, which would currently be dehydrating. Consistently with tectonic reconstructions, magmatism migration and earthquakes distribution, the subducting slab was located below the Transylvanian Basin during the Miocene and endured a rollback over the Neogene until it reached a near-vertical position beneath Vrancea. At first connected to the Tethyan suture zone, the slab got offset eastward due to limited continental delamination, possibly coeval with its verticalization. The delamination of the continental mantle, from which the offset position of the slab originates (relative to the Tethyan suture zone within the Carpathians), supports that the slab is attached to the continental lithosphere and that it is pulling on it. We suggest that the pulling force of this small slab remnant is fully counterbalanced by the resistance of the upper lithosphere. As a consequence, the slab remnant may not be currently sinking and is likely warming up at a fixed pressure since its verticalization (Fig. 2b), which would mean that all studied intermediate-depth earthquakes are due to an increase of temperature, without any additional/external change of pressure, stress or strain rate. Therefore, it may explain why the P–T diagram shows a much clearer correlation between dehydration and seismicity ("S" shape) than observed in classic subduction settings (Figs. 3 and 4).

Our results do not imply that the dehydrating slab exists only in the Carpathian corner of the Vrancea area. Instead, they show that the slab there undergoes both high stresses (elastic strain) and dehydration reactions (i.e. earthquake trigger). Stress patterns in the slab strongly suggest that the Vrancea seismic cluster would correspond to a zone of high stress concentrations associated with enhanced rock tearing^6^. The stress level is expected to be high due to both slab (un)bending and the geometrical complexity of the Carpathians, contrary to dehydration reactions, which should occur anywhere an oceanic slab is warming up. The high seismicity is located within the deep lithospheric mantle of the slab, where the hydration level is expected to be very limited, which favours earthquakes triggering consistently with the dehydration-driven stress transfer model^13^. In other words, intermediate-depth earthquakes require both dehydration reactions, which are the trigger of local mechanical instabilities, and enough elastic strain, which is eventually released seismically^12,13,19^. One could suggest that less stressed segments of the slab could extend further North, but the Ciomadul and PVF are the very last volcanic activity associated with the subduction and slab rollback, respectively, which testifies that the Vrancea slab is the very last remnant of this branch of Tethyan ocean.

Furthermore, we observe that the intermediate-depth seismicity has decreased since the 80’s (Figure S2), which is the first decade with a full recorded dataset. Comparing the total radiated energy (Mw > 2), it appears that the overall intermediate-depth seismicity has decreased since the invention of seismology, even though some large events occurred during the 70s and 80s (Figure S3), which suggests that the slab may be locked in its current position since the post-Miocene rollback. This is consistent with the idea that the main detachment process at the Carpathians scale would be achieved, with a locked slab remnant beneath Vrancea. If so, the observed intermediate-depth seismicity cluster in Vrancea should continue decreasing. Any increase in seismicity in the area in the next decades could reveal new strain localization associated with additional necking.

We further propose that the other examples of subcontinental seismic slabs (e.g.^10,47,48^) correspond to remnants of subducted slabs offset from oceanic suture zones due to substantial continental subduction. Nonetheless, in contrast with our observations beneath Romania, the seismic Hindu Kush slab, southern Asia, is demonstrably sinking^47–49^. The slab detachment process is identified at 180–265 km depth, with substantial continental subduction possibly reaching ≈ 180 km^47,49^, but the seismicity between 60 and 180 km depth likely highlights, as for the Vrancea slab, the dehydration of hydrous minerals, which should be deciphered by further investigations.

### Conclusion

The Vrancea intermediate-depth seismicity is evidence of the current dehydration of an oceanic slab beneath Romania. Most hypocentral conditions match relatively well antigorite dehydration between 2 and 4.5 GPa (60 to 135 km); at higher pressures, the dehydration of the 10-Å phase provides the best fit. More than 95 % of the dehydration-induced earthquakes are located at depths between ≈ 60 and ≈ 170 km.

Limited continental delamination due to slab pull explains the eastward offset between the Alpine Tethys oceanic suture zone and the Vrancea slab. Delaminated continental material is located between ≈ 40 and ≈ 60 km depth, which explains the seismic gap at these depths as the subcontinental mantle is not much serpentinized.

Finally, the Vrancea intermediate-depth seismicity has decreased since the invention of seismology, and we propose that the slab has been warming up at a fixed depth and may not be currently sinking.

### Methods

In this study, we consider separately the regular (< 60 km) and intermediate-depth (≥ 60 km) seismicity in the Vrancea seismic zone, consistently with previous studies. We use the regional classification of the seismic events^2^, while their primary information is extracted from the BIGSEES (http://infp.infp.ro/bigsees/Results.html) and ROMPLUS (http://www.infp.ro/index.php?i=romplus) catalogues. We select events with the minimum moment magnitude (Mw) of 2 and with depths over 10 km. The total released energy is estimated for each decade (Figure S3) based on the computation of each event energy release (E) using the classical equation:

$$E = 10.48+1.63{\times M}_{W}.$$

Information about the 3D structure of P-wave velocity beneath this region is built combining the data from the high-resolution imaging of the upper-mantle^5^ and crust^50,51^. The 3D model of Martin et al.^5^ is the most detailed P-wave velocity structure for the Vrancea slab and is adopted by many regional/local studies (e.g.^11,52–54^). To evaluate the local variations within this area, an empirical equation^55,56^ is used to derive the density (kg·m^-3^) from the P-wave velocity (${V}_{P}$, km·s^−1^): $\rho = 103 \times (0.7212+0.3209\times {V}_{P}$). The empirical pressure ($P$) is computed based on the retrieved density ($\rho$) and depth ($z$) within each grid point using the traditional equation: $P=\rho \times g\times z$, with $g=9.8$ m s^−2^.

Tomography methods provide high 3D resolution results that consist of relative variations and estimates of absolute values. Their output is highly dependent on the a-priori model and requires the integration of large amounts of data from other techniques^57^. In this study, we use the 3D thermal model of the Vrancea region^4,38,55^ based on the results of regional seismic tomography (P-waves^5^), seismic refraction studies, and the surface heat flow^38^. We combine the thermal model with the seismicity database in order to retrieve the P–T conditions for each hypocentre, which allow us to compare with experimentally deduced stability limits for a large catalogue of mineral phases expected in both continental and oceanic lithospheres (Table S1).

In addition, some uncertainties are related to the earthquake localization. The maximum and mean errors of the selected dataset are presented in Figs. 2 and 3. We observed that for some events ($\sim$ 1%) recorded before 2010, the error related to their location in 3D is large (Fig. 2) mostly due to the poor constraint of the localization due to the used 1D velocity model and the limited number of stations. Yet, their location (Figs. 2) and P–T conditions (Figs. 3) tend to follow the same trend as for other events. Relocation of these events using 3D velocity models should be done in future characterizations and interpretations of the nature of the Vrancea slab and associated seismicity.

The P–T conditions we extract for each event are associated with a number of uncertainties. First, uncertainties on P are due to uncertainties on depth estimates (see above) and ${V}_{P}$ conversion (linear relationship between $P$ and $z$). The ${V}_{P}$ errors are mostly constrained by the grid size of the tomography^5^. Concerning the resolution of the thermal model, a sensitivity analysis was performed by Ismail-Zadeh et al.^38^ to understand how stable is the numerical solution by adding small perturbations on input temperatures. Their results show that the solution is stable and the maximum temperature residual does not exceed 50 °C. In Fig. 3, we consider a mean temperature error of 25 °C.

## Supplementary Information

Supplementary Information.
