# Supplementary material for: Dehydration-induced earthquakes identified in a subducted oceanic slab beneath Vrancea, Romania
Source: Sci Rep. 2021 May 13;11:10315. doi: 10.1038/s41598-021-89601-w (PMC8119720; doi:10.1038/s41598-021-89601-w)
Supplement: Supplementary file 1 — Supplementary Information. [file 41598_2021_89601_MOESM1_ESM.pdf]

Supplementary Material – Dehydration-induced earthquakes identified in a subducted oceanic slab beneath Vrancea, Romania

Thomas P. Ferrand[1,*], Elena F. Manea[2,3]

1: Institut des Sciences de la Terre d'Orléans, CNRS UMR 7327, Université d'Orléans, France.

2: National Institute for Earth Physics, Calugareni, 12, Măgurele Ilfov, Romania.

3: GNS Science, PO Box 30-368, Lower Hutt, New Zealand

*Corresponding author: thomas.ferrand@univ-orleans.fr

Table S1: Database of stability limits for hydrous minerals that are likely within the Vrancea slab.

Group	Name	abbrv.	Formula	Context	References
Hydroxide	Brucite	Br	$Mg(OH)_2$	mantle	Berman et al., 1986
Serpentines	Chrysotile	Chr	$(Mg,Al)_3(Si,Al)_2O_5(OH)_4$		Ulmer & Trommsdorff, 1999; Schwartz et al. 2013
	Lizardite	Lz			
	Antigorite	Atg	$Mg_{48}Si_{34}O_{85}(OH)_{62}$ $m = 17$; $(Mg,Al)_{3m-3}(Si,Al)_{2m}O_{5m}(OH)_{4m-6}$ $m = [\![13;24]\!]$		Wunder & Schreyer, 1997; Ulmer & Trommsdorff, 1999; Bromiley & Pawley, 2003; Evans, 2004; Perrillat et al., 2005; Hilairet et al., 2006; review: Ferrand, 2019a
Amphiboles	Hornblende	Hb	$\square(K,Na,Ca)_2[Mg_4Al][Si_7Al]O_{22}(OH)_2$	crust	Poli & Schmidt., 2002
	Mg-katophorite	Kat	$\square Na_2CaMg_4Al_2Si_3O_{11}[Si_4O_{11}](OH)_2$		Pirard & Hermann, 2015
	K-richterite	Ri	$\square KNaCaMg_5[Si_4O_{11}]_2(OH)_2$		Gilbert & Briggs, 1974
	Glaucophane	Gln	$\square Na_2[Mg_3Al_2][Si_4O_{11}]_2(OH)_2$		Carman & Gilbert, 1983; Guiraud et al., 1990; Tropper et al., 2000; Incel et al., 2017
	Pargasite	Prg	$\square NaCa_2[Mg_4Al][Si_6Al_2]O_{22}(OH)_2$	crust / mantle	review: Pirard & Hermann, 2015
	Tremolite	Tr	$\square Ca_2Mg_5[Si_4O_{11}]_2(OH)_2$	mantle	Ulmer & Trommsdorff, 1999
	Anthophyllite	Ant	$\square Mg_2Mg_5[Si_4O_{11}]_2(OH)_2$		Berman et al., 1986
	Cummingtonite	Cum	$\square Ca_xMg_{2-x}Mg_5[Si_4O_{11}]_2(OH)_2$ $(0 < x < 1)$		Evans & Ghiorso, 1995
Sorosilicates	Lawsonite	Law	$CaAl_2[Si_2O_7](OH)_2 \bullet H_2O$	crust	Holland & Powell, 1990; Schmidt & Poli, 1998; Incel et al., 2017; review: Martin et al., 2014
	Zoisite	Zo	$Ca_2Al_3[SiO_4][Si_2O_7]O(OH)$		Poli & Schmidt, 1998; 2002; Poli, 2016
	Epidote	Ep	$Ca_2(Fe,Al)Al_2[SiO_4][Si_2O_7]O(OH)$		
Tourmaline		Trm	$(\square,Na,K...)(Mg,Al...)_3Al_6[Si_6O_{18}][BO_3]_3(OH)_4$	crust / mantle	review: van Hinsberg et al., 2011
	Mg-foitite	Ft	$(\square,Na)[Mg_2Al]Al_6[Si_6O_{18}][BO_3]_3(OH)_4$		Werding & Schreyer, 1984
	Dravite	Drv	$NaMg_3Al_6[Si_6O_{18}][BO_3]_3(OH)_4$		Robbins & Yoder, 1962; Krosse, 1995; Ota et al., 2008
Mica	Aspidolite		$NaMg_3[AlSi_3O_{10}](OH)_2$	mantle	Carman & Gilbert, 1983
	Phengite	Phe	$KAl_{1.5}(Mg,Fe)_{0.5}[Al_{0.5}Si_{3.5}O_{10}](OH)_2$	crust	Chopin, 1984; Poli & Schmidt, 2002
	Paragonite	Para	$NaAl_2[AlSi_3O_{10}](OH)_2$		Guiraud et al., 1990; Droop, 2013
Talc & Hydrated talc	Talc	Ta	$(Mg,Al)_3(Si, Al)_4O_{10}(OH)_2$; $Mg_3Si_4O_{10}(OH)_2$	crust / mantle	Chopin, 1984; Guiraud et al., 1990; Kithara et al., 1966; Chopin, 1984; Berman et al. 1986; Pawley & Wood, 1995; Yamamoto & Akimoto, 1977; Ulmer & Trommsdorff, 1999; Bailey & Holloway, 2000; review: Ferrand, 2019
	10-Å phase	10Å	$(Mg,Al)_3(Si,Al)_4O_{10}(OH)_2 \bullet x H_2O$ $(0 < x \le 2)$		Chinnery et al., 1999; Chollet et al., 2009; Wunder & Schreyer, 1997

Table S1 (continued)

Group	Name	abbrv.	Formula	Context	References
Chlorites	Clinochlore	Chl	$Mg_5Al_2Si_3O_{10}(OH)_8$	crust	Apted & Liou, 1983<br>Poli & Schmidt, 2002
				mantle	Pawley, 2003<br>Staudigel & Schreyer, 1977
	Sudoite	Sud	$Mg_2Al_4Si_3O_{10}(OH)_8$	mantle	Fransolet & Schreyer, 1984
Chloritoid	Mg-chloritoid		$Mg_2Al_4Si_2O_{10}(OH)_4$	mantle	Chopin & Schreyer, 1983
Humites	Ti-clinohumite	CH	$[Mg_2SiO_4]_4Mg_{1-x}Ti_xO_{2x}(OH,F)_{2-2x}$ $(0 \le x < 1)$<br>$[Mg_2SiO_4]_4Mg_{0.5}Ti_{0.5}O(OH)$	mantle	Weiss, 1997
DHMS	Phase A	A	$[Mg_2SiO_4]_2Mg_3(OH)_6$	mantle	Pawley & Wood, 1996
Pumpellyites & Sursassites	Pumpellyite	Pmp	$Ca_2MgAl_2[SiO_4][Si_2O_7](OH)_2 \bullet H_2O$	crust	Liou, 1971
			$Ca_2Mg_{0.5}Al_{2.5}[SiO_4][Si_2O_7](OH)_2 \bullet H_2O$	crust	Schiffman & Liou, 1980
	Mg-sursassite	Sur	$[MgAl]_5[Si_2O_7]_3(OH)_7$	mantle	Fockenberg, 1998b
Staurolite	Mg-staurolite	Stau	$Mg_2Al_9[SiO_4]_4O_7(OH)$	mantle	Fockenberg, 1998a
Carpholite	Mg-carpholite	Car	$MgAl_2[Si_2O_7](OH)_2 \bullet H_2O$	crust	Guiraud et al., 1990
				mantle	Chopin & Schreyer, 1983
Prehnite		Prh	$Ca_2Al(Si,Al)_4O_{10}(OH)_2$	crust	Liou, 1971
Zeolites	Wairakite	Wrk	$CaAl_2Si_4O_{12} \bullet 2\ H_2O$	crust	Liou, 1970
Anhydrous phases associated with dehydration reactions	Forsterite	Fo	Mg_2SiO_4	mantle	
	Enstatite	En	$Mg_2Si_2O_6$	mantle<br>crust	
	Jadeite	Jd	$NaAlSi_2O_6$	crust	
	Anorthite	An	$CaAl_2Si_2O_8$	crust	
	Kyanite	Ky	Al_2SiO_5	crust	
	Quartz	Qz	SiO_2	crust	

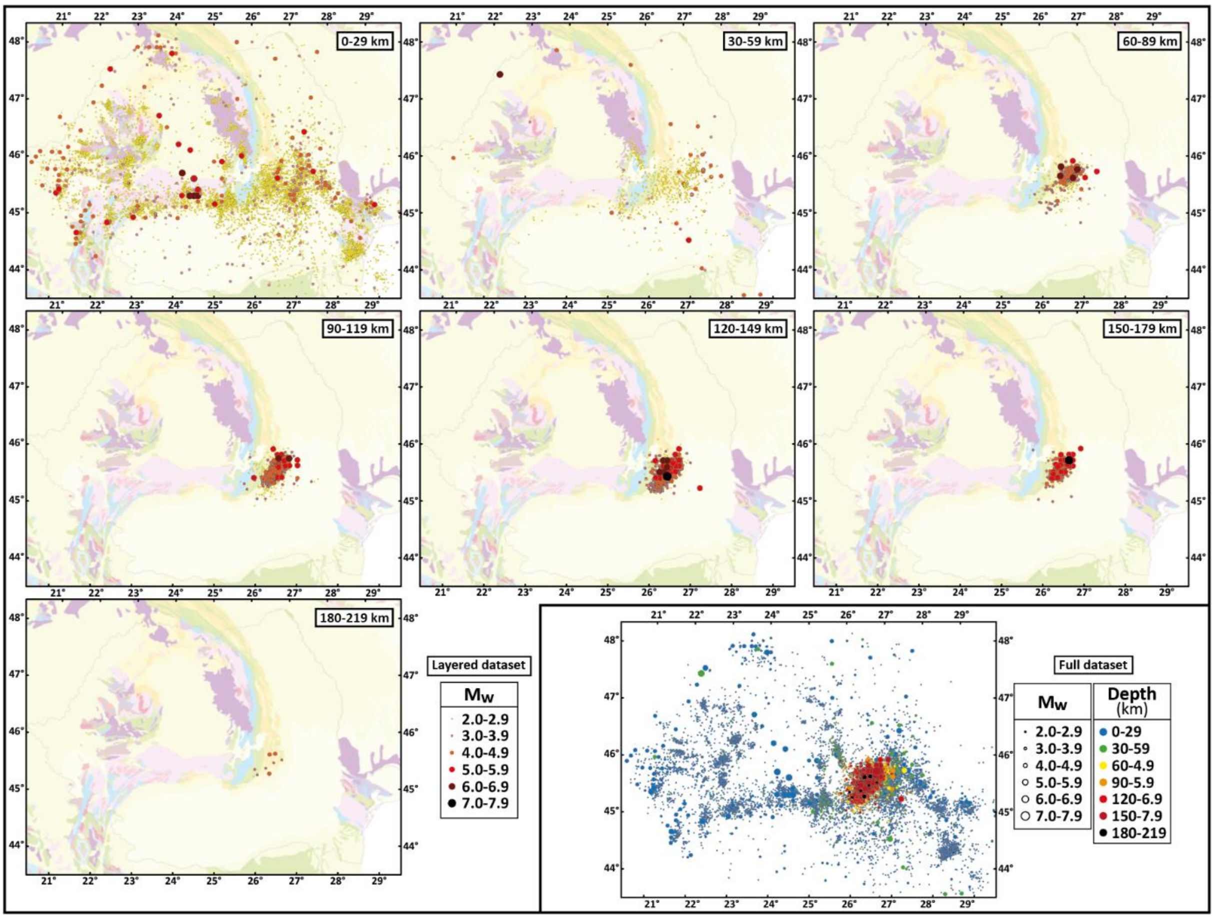

Figure S1: Detailed view of the seismicity distribution in the SE Carpathians. The full dataset for the period 1940-2019 is presented, for events with Mw ≥ 2. The seismicity is shown in 30-km-thick layers, overlapping the geological map. The entire dataset is summarized in the bottom right panel. Maps generated with *ArcGIS 10.6* using a basemap from Asch (2003) together with the *BIGSEES* and *ROMPLUS* catalogues (see ***Methods***).

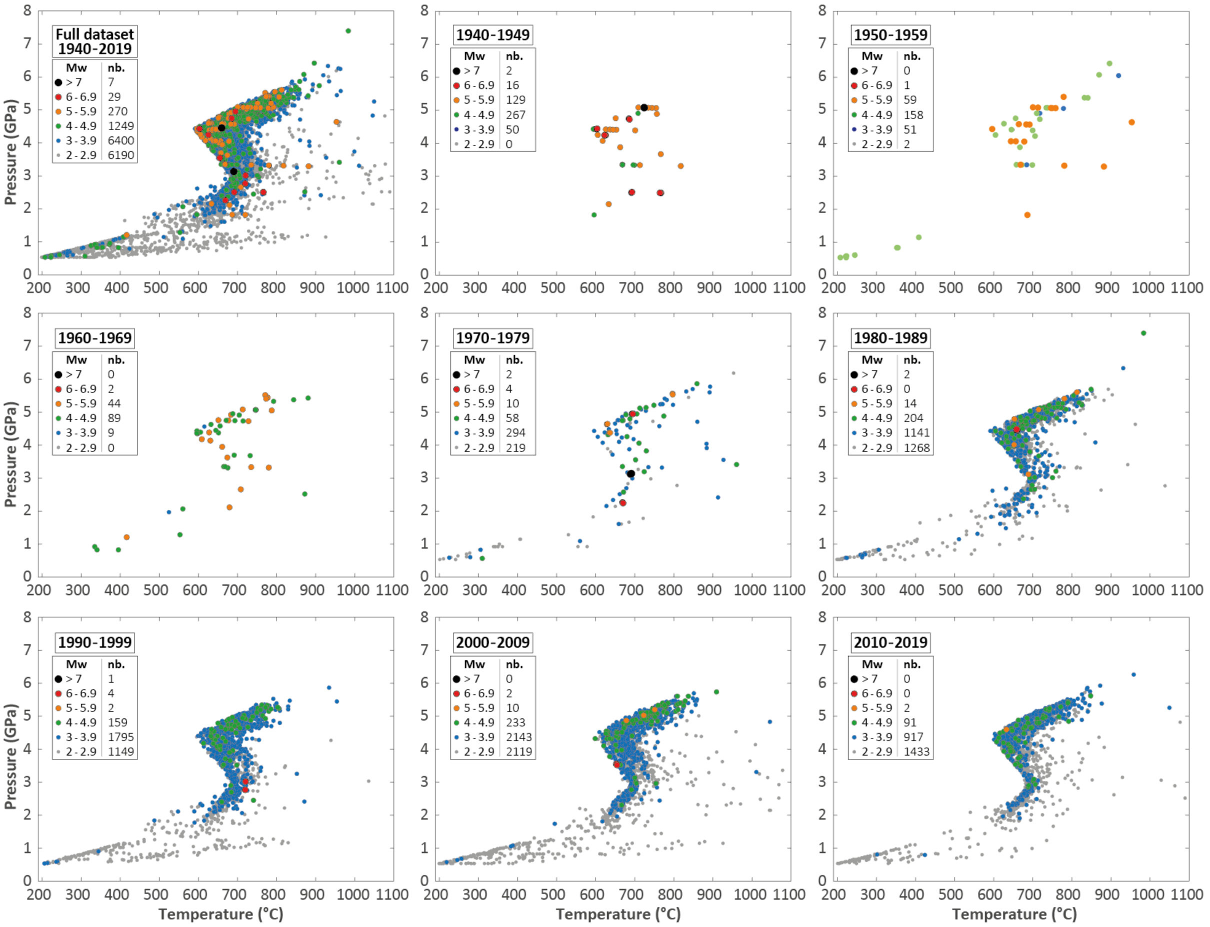

Figure S2: Evolution of the Vrancea seismicity through time. P-T conditions computed for hypocenters based their location within and around the slab. The full dataset (Mw ≥ 2; 1940-2019) is presented in the top-left subfigure. The other subfigures show the situation decade by decade.

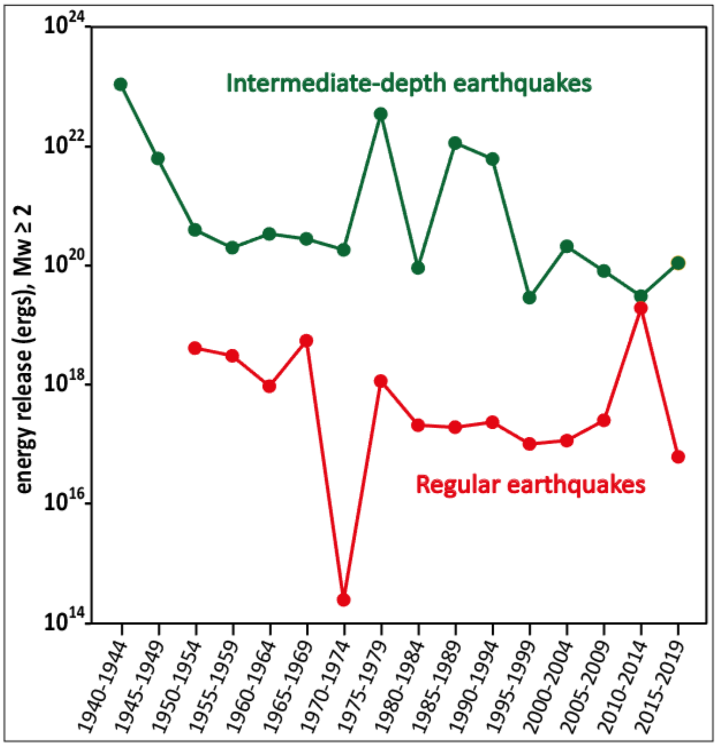

Figure S3: Total seismic energy release through time. Energy calculation as described in the methods, for both intermediate-depth (≥ 60 km depth) and regular (< 60 km) earthquakes.

Supplementary references

1. Apted, M. J. & Liou, J. G. 1983. Phase relations among greenschist, epidote-amphibolite, and amphibolite in a basaltic system. *American Journal of Science* **283**(A), 328-354.
2. Asch, K. 2003. The 1: 5 million international geological map of Europe and adjacent areas: Development and Implementation of a GIS-enabled Concept. Geologisches Jahrbuch, SA 3, Stuttgart: E. Schweizerbartsche Verlagsbuchhandlung.
3. Bailey, E. & Holloway, J.R. 2000. Experimental determination of elastic properties of talc to 800 C, 0.5 GPa; calculations of the effect on hydrated peridotite, and implications for cold subduction zones. *Earth & Planetary Science Letters* **183**(3-4), 487-498.
4. Berman, R. G. 1988. Internally-consistent thermodynamic data for minerals in the system Na_2O-K_2O-CaO-MgO-FeO-Fe_2O_3-Al_2O_3-SiO_2-TiO_2-H_2O-CO_2. *Journal of petrology* **29**(2), 445-522.
5. Bromiley, G.D. & Pawley, A.R. 2003. The stability of antigorite in the systems MgO-SiO_2-H_2O (MSH) and MgO-Al_2O_3-SiO_2-H_2O (MASH): the effects of Al3+ substitution on high-pressure stability. *American Mineralogist* **88**(1), 99-108.
6. Chinnery, N.J., Pawley, A.R. & Clark, S.M. 1999. In situ observation of the formation of 10-Å phase from talc + H2O at mantle pressures and temperatures. *Science* **286**(5441), 940-942.
7. Chollet, M., Daniel, I., Koga, K.T., Petitgirard, S. & Morard, G. 2009. Dehydration kinetics of talc and 10-Å phase: consequences for subduction zone seismicity. *Earth & Planetary Science Letters* **284**(1-2), 57-64.
8. Chopin, C. & Schreyer, W. 1983. Magnesiocarpholite and magnesiochloritoid: two index minerals of pelitic blueschists and their preliminary phase relations in the model system $MgOAl_2O_3$-SiO_2-H_2O. *American Journal of Science **283**, 72-96.*
9. Droop, G. T. R. 2013. Paragonite in marbles from the Tauern Window, Austria: Compositional and thermobaric controls. *Lithos* **162**, 1-13.
10. Evans, B.W. & Ghiorso, M.S. 1995. Thermodynamics and petrology of cummingtonite. *American Mineralogist* **80**(7-8), 649-663.
11. Evans, B. W. 2004. The serpentinite multisystem revisited: chrysotile is metastable. *International Geology Review* **46**(6), 479-506.
12. Fumagalli, P., Stixrude, L., Poli, S. & Snyder, D. 2001. The 10-Å phase: a high-pressure expandable sheet silicate stable during subduction of hydrated lithosphere. *Earth & Planetary Science Letters* **186**(2), 125-141.
13. Fockenberg, T. 1998a. An experimental investigation on the P-T stability of Mg-staurolite in the system MgO-Al2O3-SiO2-H2O. *Contributions to Mineralogy & Petrology* **130**(2), 187-198.
14. Fockenberg, T. 1998b. An experimental study of the pressure-temperature stability of MgMgAl-pumpellyite in the system MgO-Al_2O_3-SiO_2-H_2O. *American Mineralogist* **83**(3-4), 220-227.
15. Fransolet, A.M. & Schreyer, W. 1984. Sudoite, di/trioctahedral chlorite: a stable low temperature phase in the system MgO-Al2O3-SiO2-H2O. *Contributions to Mineralogy & Petrology* **86**(4), 409-417.
16. Guiraud, M., Holland, T. & Powell, R. 1990. Calculated mineral equilibria in the greenschist-blueschist-eclogite facies in Na_2O-FeO-MgO-Al_2O_3-SiO_2-H_2O. *Contributions to Mineralogy & Petrology* **104**(1), 85-98.
17. Hilairet, N., Daniel, I. & Reynard, B. 2006. Equation of state of antigorite, stability field of serpentines, and seismicity in subduction zones. *Geophysical Research Letters* **33**(2).
18. van Hinsberg, V. J., Henry, D. J. & Marschall, H. R. 2011. Tourmaline: an ideal indicator of its host environment. *The Canadian Mineralogist* **49**(1), 1-16.
19. Holland, T. J. B. & Powell, R. 1990. An enlarged and updated internally consistent thermodynamic dataset with uncertainties and correlations: the system K_2O-Na_2O-CaO-MgO-MnO-FeO-Fe_2O_3-Al_2O_3-TiO_2-SiO_2-C-H_2-O_2. *Journal of metamorphic Geology* **8**(1), 89-124.
20. Kithara, S., Takenouchi, S. & Kennedy, G.C. 1966. Phase relations in the system $MgOSiO_2$-H_2O at high-temperatures and pressures. *American Journal of Science* **264**, 223-233.
21. Krosse, S. 1995. Hochdruckssyntheses, Stabilität und Eigenschaften der Borsilikate Dravit und Kornerupin sowie Darstellung und Stabilitätsverhalten eines neuen Mg-Al-borates. Doctor thesis, Ruhr-Universität, Bochum.
22. Liou, J. G. 1971. Synthesis and stability relations of prehnite, $Ca_2Al_2Si_3O_{10}(OH)_2$. *American Mineralogist: Journal of Earth & Planetary Materials* **56**(3-4), 507-531.
23. Liou, J. G. 1970. Synthesis and stability relations of wairakite, $CaAl_2Si_4O_{12} \cdot 2\ H_2O$. *Contributions to Mineralogy & Petrology* **27**(4), 259-282.

24. Martin, L. A. J., Hermann, J., Gauthiez-Putallaz, L., Whitney, D. L., Vitale Brovarone, A., Fornash, K. F. & Evans, N. J. 2014. Lawsonite geochemistry and stability–implication for trace element and water cycles in subduction zones. *Journal of Metamorphic Geology* **32**(5), 455-478.
25. Ota, T., Kobayashi, K., Katsura, T. & Nakamura, E. 2008. Tourmaline breakdown in a pelitic system: implications for boron cycling through subduction zones. *Contributions to Mineralogy & Petrology* **155**(1), 19-32.
26. Pawley, A. 2003. Chlorite stability in mantle peridotite: the reaction clinochlore + enstatite = forsterite + pyrope + H2O. *Contributions to Mineralogy & Petrology* **144**(4), 449-456.
27. Pawley, A.R. & Wood, B.J. 1996. The low-pressure stability of phase A, $Mg_7Si_2O_8(OH)_6$. *Contributions to Mineralogy & Petrology* **124**, 90-97.
28. Pawley, A.R. & Wood, B.J. 1995. The high-pressure stability of talc and 10- Å phase: potential storage for H2O in subduction zones. *American Mineralogist* **80**, 998-1003.
29. Perrillat, J. P., Daniel, I., Koga, K. T., Reynard, B., Cardon, H. & Crichton, W. A. 2005. Kinetics of antigorite dehydration: a real-time X-ray diffraction study. *Earth & Planetary Science Letters* **236**(3-4), 899-913.
30. Pirard, C. & Hermann, J. 2015. Experimentally determined stability of alkali amphibole in metasomatised dunite at sub-arc pressures. *Contributions to mineralogy & petrology* **169**(1), 1.
31. Poli, S. 2016. Melting carbonated epidote eclogites: carbonatites from subducting slabs. *Progress in Earth & Planetary Science* **3**(1), 27.
32. Poli, S. & Schmidt, M. W. 2002. Petrology of subducted slabs. *Annual Review of Earth & Planetary Sciences* **30**(1), 207-235.
33. Poli, S. & Schmidt, M. W. 1998. The high-pressure stability of zoisite and phase relationships of zoisite-bearing assemblages. *Contributions to Mineralogy & Petrology* **130**(2), 162-175.
34. Robbins, C. R. & Yoder Jr, H. S. 1962. Stability relations of dravite, a tourmaline. *Carnegie Institution of Washington, Yearbook* **61**, 106-108.
35. Schiffman, P. & Liou, J. G. 1980. Synthesis and Stability Relations of Mg-Al Pumpellyite, $Ca_4Al_5MgSi_6O_{21}(OH)_7$. *Journal of Petrology* **21**(3), 441-474.
36. Schmidt, M. W. & Poli, S. 1998. Experimentally based water budgets for dehydrating slabs and consequences for arc magma generation. Earth & Planetary Science Letters 163(1-4), 361-379.
37. Schwartz, S., Guillot, S., Reynard, B., Lafay, R., Debret, B., Nicollet, C., Lanari, P. & Auzende, A.L. 2013. Pressure-temperature estimates of the lizardite/antigorite transition in high pressure serpentinites. *Lithos* **178**, 197-210.
38. Staudigel, H. & Schreyer, W. 1977. The upper thermal stability of clinochlore, $Mg_5Al[AlSi_3O_{10}](OH)_8$, at 10-35 kb PH_2O. *Contributions to Mineralogy & Petrology* **61**(2), 187-198.
39. Tropper, P., Manning, C. E., Essene, E. J. & Kao, L. S. 2000. The compositional variation of synthetic sodic amphiboles at high and ultra-high pressures. *Contributions to Mineralogy & Petrology* **139**(2), 146-162.
40. Ulmer, P. & Trommsdorf. V. 1999. Phase relations of hydrous mantle subducting to 300 km. Mantle petrology. In *Mantle Petrology: Field Observations & High-Pressure Experimentation*. Geochemical Society, 259-281.
41. Weiss, M. 1997. Clinohumites: A Field and Experimental Study. Doctoral dissertation. *Swiss Federal Institute of Technology Zurich.*
42. Werding, G. & Schreyer, W. 1984. Alkali-free tourmaline in the system $MgO-Al_2O_3-B_2O_3-SiO_2-H_2O$. *Geochimica et Cosmochimica Acta* **48**(6), 1331-1344.
43. Wunder, B., & Schreyer, W. 1997. Antigorite: High-pressure stability in the system $MgO-SiO_2-H_2O$ (MSH). *Lithos* **41**(1-3), 213-227.
44. Yamamoto, K., Akimoto, S.I. 1977. The system $MgO-SiO_2-H_2O$ at high pressures and temperatures; stability field for hydroxyl-chondrodite, hydroxyl-clinohumite and 10-Å phase. *American Journal of Science* **277**(3), 288-312.
